# Inotuzumab ozogamicin in clinical development for acute lymphoblastic leukemia and non-Hodgkin lymphoma

**DOI:** 10.1186/s40364-019-0160-4

**Published:** 2019-04-11

**Authors:** Amandeep Aujla, Ravijot Aujla, Delong Liu

**Affiliations:** 1 Department of Medicine, New York Medical College and Westchester Medical Center, Valhalla, NY 10595 USA; 2 Punjab Institute of Medical Sciences, Jalandhar, Punjab 144006 India; 3 Department of Oncology, The First affiliated hospital of Zhengzhou University, Zhengzhou, China

**Keywords:** Acute lymphoblastic leukemia, CD22, Inotuzumab ozogamicin, Non-Hodgkin lymphoma, Antibody-drug conjugate, ADC

## Abstract

B cell acute lymphoblastic leukemia (ALL) and non-Hodgkin lymphoma (NHL) frequently express CD19, CD20 and CD22 on the cell surfaces. Immunotherapeutic agents including antibodies and chimeric antigen receptor T cells are widely studied in clinical trials. Several antibody-drug conjugates (ADC) have been approved for clinical use (gemtuzumab ozogamicin in acute myeloid leukemia and brentuximab vedotin in Hodgkin lymphoma as well as CD30+ anaplastic large cell lymphoma). Inotuzumab ozogamicin (INO), a CD22 antibody conjugated with calicheamicin is one of the newest ADCs. INO has been approved for treatment of relapsed /refractory B cell precursor ALL. Multiple ongoing trials are evaluating its role in the relapsed /refractory B cell NHL. This review summarized recent development in INO applications for ALL and NHL.

## Introduction

The prognosis of adults with relapsed /refractory (R/R) acute lymphoblastic leukemia (ALL) and non-Hodgkin lymphoma (NHL) is still very poor [1–5]. With the increasing use of next-generation sequencing (NGS) and molecular biomarkers, early diagnosis and targeted therapy become possible [6–10]. It appears that NGS-based minimal residue disease (MRD) monitoring may better predict MRD relapse and lead to earlier intervention [11]. B cell ALL and NHL frequently express CD19, CD20 and CD22 on the cell surfaces. Monoclonal antibodies (MoAb) against CD20 have been widely used for the treatment of multiple lymphoid malignancies [12–14]. Immunotherapy with bispecific antibodies such as blinatumomab against CD19 is being studied in multiple types of B cell malignancies [15–23]. Immune checkpoint inhibitors have also been approved for treatment of Hodgkin lymphoma [24]. Chimeric antigen receptor (CAR) T cells are also being widely studied in clinical trials [25–31]. CD19 is the most commonly targeted surface marker in CAR T trials [32–37]. CD20, CD22, and CD30 are also targeted antigens of CAR T cells in ALL and lymphoma trials [26]. Tisagenlecleucel has been approved for R/R B ALL and diffuse large B cell lymphoma (DLBCL) [36, 38–42]. In addition, axicabtagene ciloleucel has been approved for R/R DLBCL [43, 44].

In addition to the above immunotherapeutic agents, conjugation of cytotoxic agents with monoclonal antibodies is an evolving field with the development of multiple targeted cytotoxic agents called antibody-drug conjugates (ADC) [45]. These are being used and studied with targets across different malignancies (e.g. trastuzumab emtansine in breast cancer, gemtuzumab ozogamicin in acute myeloid leukemia and brentuximab vedotin in Hodgkin lymphoma as well as CD30+ anaplastic large cell lymphoma) [46–52]. Inotuzumab ozogamicin (INO), a CD22 MoAb conjugated with calicheamicin is one of the newest ADCs in clinical application [53, 54]. INO has been approved for treatment of R/R B cell precursor ALL [55–63]. Multiple ongoing trials are evaluating its role in the R/R B cell NHL. This review summarized recent development in INO applications for B cell ALL and NHL.

## CD22 expression and function

**Fig. 1:**
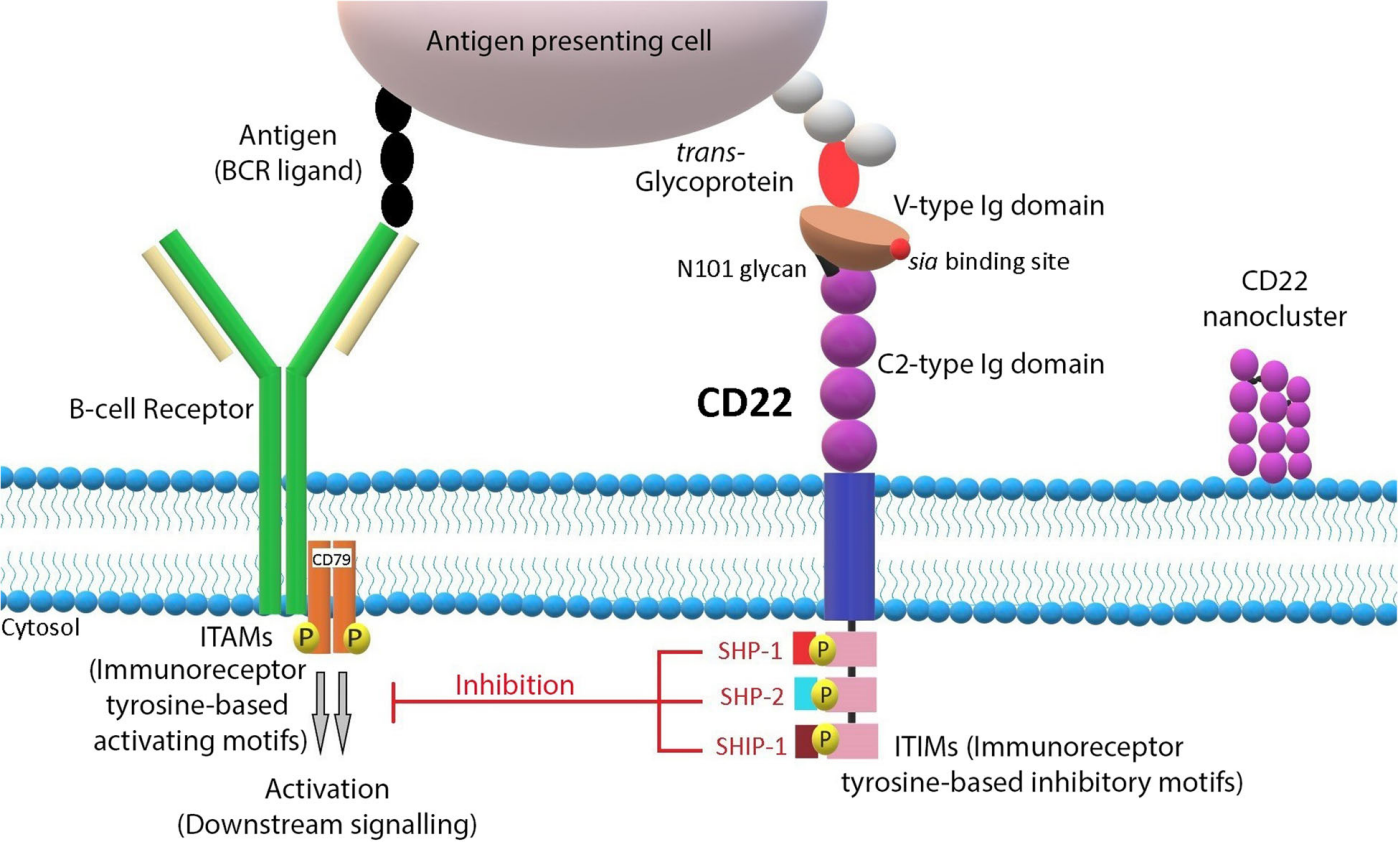
CD22 structure and signaling pathway. CD22 molecule is a transmembrane protein from SIGLEC (Sialic acid-binding immunoglobulin-type lectins) family. It has three parts: (i) V-type Ig domain with *sia*-binding site, (ii) C1-type Ig domain, (iii) C2-type Ig domain. The CD22 intracellular region contains ITIMs (Immunoreceptor tyrosine-based inhibitory motifs). The tyrosine residues of the ITIMs become phosphorylated with the ligand binding, which leads to activation of SHP-1 (*Src* homology region 2 domain-containing phosphatase-1), SHP-2 (*Src* homology region 2 domain-containing phosphatase-2), SHIP-1 ((*Src* homology region 2 domain-containing inositol 5′ polyphosphatase-1). These phosphatases act as negative regulators for down-streaming signaling from B-cell receptors

CD22 is an inhibitory component of the B-cell receptor (BCR) complex expressed exclusively in pre-B, immature and mature B cells but is lost upon differentiation to plasma cells [64–66]. It mediates negative impact on BCR signaling pathway by dephosphorylating the associated cascade components via protein tyrosine phosphatases [67–69] (Fig. 1).

## CD22 positivity in lymphoid malignancies

CD22 expression increases progressively along the pathway of B cell maturation. Raponi et al. reported CD22 expression among different subtypes of ALL as 83% of Pro-B, 96.4% of common B cell, 91.9% of Pre-B and 100% of the mature B cell ALL [70]. In mature B cell lymphoma, its expression has been reported as 95% in chronic lymphocytic leukemia, 89% in diffuse large B-cell lymphoma, 98% in follicular lymphoma, 96% in lymphoplasmacytic lymphoma and 100% in hairy cell, mantle cell, marginal zone, splenic marginal zone lymphomas and monoclonal B-lymphocytosis [71]. Hence CD22 can serve as a good target for therapy of B cell malignancies.

## Inotuzumab ozogamicin: CD22 antibody-drug conjugate

### Structure and function

Inotuzumab is a humanized IgG4 monoclonal antibody that binds CD22. It is conjugated via an acid labile linker to the cytotoxic chemotherapy, calicheamicin (N-acetyl-γ-calicheamicin dimethyl hydrazide). Calicheamicin is a potent cytotoxic antibiotic that binds DNA in the minor groove and causes double-strand DNA breaks leading to cell death [72]. Binding of drug to CD22 receptor leads to its endocytosis and cytotoxic chemotherapy is released in acidic lysosomal environment with degradation of the linker. CD22 receptor is then recycled back to the surface and may play a role in augmented efficacy [73, 74].

### Preclinical studies

CD22 monoclonal antibody (MoAb) with or without conjugation to calicheamicin has similar affinity to CD22 receptors on human B-lymphoma cells [73]. In vitro studies showed enhancement of cytotoxic potency of calicheamicin by 1.5 to 39-fold when conjugated to CD22 MoAb against CD22^+^ B-lymphoma cell lines. INO (CMC-544) was noted to inhibit the growth of CD22^+^ human B-cell lymphomas grafted subcutaneously into the mice in a dose dependent manner. Half-life of CMC-544 is 35 h and was noted to be similar in both tumor bearing and non-tumor bearing mice. However AUC (area under curve) of serum levels in tumor bearing mice was noted to be 37% lower, suggesting absorption by targeted tumor tissue [73]. Similar preclinical studies in mice with ALL cells and subcutaneous xenografts also showed that INO not only inhibited the growth of ALL xenografts but also prevented engraftment of ALL cells and development of disseminated disease in SCID (severe combined immune deficiency) mice [75, 76]. These results were also replicated in pediatric B-ALL cells with additional findings that efficacy (inducing apoptosis) is not dependent on CD22 expression and receptor saturation, in contrast to gemtuzumab ozogamicin [77]. High expression of CD22 was reported to accelerate the response in comparison to low CD22 expression cell lines.

### Clinical trials of inotuzumab ozogamicin in ALL

Phase 1 dose finding study for Inotuzumab ozogamicin (INO) in CD22-positive R/R ALL was done with 1.2, 1.6, or 1.8 mg/m2 doses per cycle on days 1, 8, and 15 over a 28-day cycle [78]. The recommended phase 2 dose (RP2D) was determined to be 1.8 mg/m2 (Table 1).

**Table 1:** Clinical trials of inotuzumab ozogamicin (INO)

Reference	Phase	Disease	Intervention INO +	ORR (CR)	mPFS	mOS	Significant toxicities
[55]	2	R/R Ph-Negative CD22 positive ALL	Mini-Hyper-CVD with INO and Rituximab	ORR was 78% (59% CR) MRD negative rates of 52% (at time of morphological response) and 82% (at 3 months).	Median RFS of 8 months.	11 months	VOD (15%); prolonged thrombocytopenia (81%); 95% suffered hepatotoxicity (20% with grade 3 or higher)
[63]	3	Refractory or Relapsed ALL	0.8 mg/m2 (D1), 0.5 mg/m2 (D8), 0.5 mg/m2 (D15) Versus Standard therapy	CR + CRi 80.7% (CR 35.8%)	5 months	7.7 months	Grade 3 or more thrombocytopenia, hepatotoxicity and VOD (11%)
[78]	1/2	R/R ALL	1.8 mg/m2 weekly	69% CR/CRi (29% CR)			cytopenias and liver toxicity
[84]	1	R/R FL (100%)	Single agent 1.3 mg/m2 q28d with dose escalation up to MTD 1.8 mg/m2 q28d	CR: 54% ORR: 85%	–	–	No DLTs. MTD of 1.8 mg/m2 confirmed in Japanese population.
[85]	1/2	CD20 and CD22 positive B-NHL. Relapsed follicular lymphoma (35%), Relapsed diffuse large B-cell lymphoma (39%), or refractory aggressive NHL (25%)	Dose escalation (0.8, 1.3 and 1.8 mg/m2) study in combination with Rituximab 375 mg/m2 MTD of determined to be 1.8 mg/m2.	FL: 87% (62%) DLBCL: 74% (50%) Refractory: 20%	FL: NR (2 year PFS rate of 68%) DLBCL: 17.1 months Refractory: 1.9 months.	FL: 2 year OS rate 90% DLBCL: 3 year OS rate 69% Refractory: 8.8 months	Grade 3 to 4 thrombocytopenia (31%) and neutropenia (22%). SAEs of Pneumonia (4%), Sepsis (3%) and liver dysfunction (4%). No VOD.
[86]	1	B-NHL (CD20 and CD22-positive, B-cell NHL which has progressed after 1 or 2 prior therapies)	1.8 mg/m2, IV on day 2 of each 28 day cycle; up to 8 cycles + R 375 mg/m2, IV on day 1 of each 28 day cycle; up to 8 cycles	80% (60%)	NR	NR	90% SAEs, with thrombocytopenia, neutropenia, elevated liver enzymes and hypophosphatemia
[87]	1	CD22 positive NHL with at least 1 prior treatment	INO (0.8 mg/m2) + RCVP	84% (24%)	14.4 months	24.5 months	1 death due to neutropenic pneumonia in INO-CVP arm. (13/48) 27% discontinued therapy in INO-CVP arm due to adverse effects
[88]	1/2	CD22 positive NHL with at least 1 prior treatment; DLBCL (38%) FL (25%) MCL (24%) Refractory (42%)	INO (0.8 mg/m2) + R- GDP	Phase 1: 53% (20%);	6 m: 58% 12 m: 37% 24 m: 24%	6 m:81% 12 m: 61% 24 m: 55%	Grade 3 or more thrombocytopenia (75%); neutropenia (62%). One patient with grade 3 VOD.
				Phase 2 dose (RP2D): 50% (14%)			
				Refractory: 35%			

*Abbreviations*: *R/R* refractory /relapsed, *CVD* cyclophosphamide vincristine dexamethasone, *m* month, *ORR* overall response rate, *CR* complete remission, *PFS* progression free survival, *OS* overall survival, *RFS* relapse free survival, *VOD* veno-occlusive disease, *NHL* non-Hodgkin lymphoma, *NR* not reached, *MRD* minimal residual disease, *MTD* maximal tolerated dose, *SAE* serious adverse event, *DLBCL* diffuse large B cell lymphoma, *FL* follicular lymphoma, *MCL* mantle cell lymphoma, *RP2D* recommended phase 2 dose, *GDP* gemcitabine dexamethasone cisplatin

The safety and efficacy of INO were further assessed in phase 2 expansion cohort. INO was given as 0.8 mg/m2 on day 1; 0.5 mg/m2 on days 8 and 15; The dosage was lowered to 1.6 mg/m2 per cycle after complete remission (CR) or CR with incomplete marrow recovery (CRi). CR/CRi was achieved in 69% (CR 29%) with RP2D and MRD negativity was reported in 75% of this population (CR/CRi). Median progression free survival (PFS) in all treated population was 3.9 months and median overall survival (OS) of 7.4 months. Twenty-four out of 72 (33%) patients in total proceeded to allogeneic stem cell transplant (AlloSCT) and most of the patients were given fludarabine and/or total body irradiation (TBI) based conditioning regimen except one patient who received dual alkylator conditioning (cyclophosphamide, thiotepa, and fludarabine). Among these, 12 deaths occurred (2 died due to relapse/progressive disease; 7 died ≤100 days due to sepsis, graft-versus-host disease, venoocclusive disease and respiratory failure). Four patients developed venoocclusive disease (VOD), none of whom had received pre-study AlloSCT (Two patients experienced VOD during therapy or follow-up without AlloSCT and two developed VOD after AlloSCT) [78].

INO has been approved by FDA for treatment of adults with R/R B-cell precursor ALL based on results of INO-VATE trial [63]. This phase 3 trial compared INO given as 0.8 mg/m2 on day (D)1 followed by 0.5 mg/m2 on D8 and D15 (total 1.8 mg/m2 every 4 weeks) against standard chemotherapy in Ph-positive or Ph-negative refractory or relapsed B-ALL. Chemotherapy regimens included FLAG (Fludarabine, cytarabine and Granulocyte stimulating factor), cytarabine with mitoxantrone or cytarabine alone. This phase 3 study demonstrated that single agent INO led to a significantly higher CR rate than that in the chemotherapy group (80.7% vs. 29.4%; *p* < 0.001), and a longer CR duration (4.6 vs. 3.1 months; *p* = 0.03).

Veno-occlusive disease (VOD) with liver function abnormality and weight gain was a major adverse event. Therefore, careful planning for INO therapy prior to AlloSCT is important to minimize VOD complications. It is generally advised that length of INO therapy should be limited. Longer spacing from end of INO therapy to AlloSCT is also being studied, such as adding blinatumomab as consolidation prior to AlloSCT [79, 80].

INO has also been studied in a phase 2 trial in combination with chemotherapy for R/R Philadelphia chromosome-negative ALL [55]. It was combined with mini-Hyper-CVD regimen (miniHCVD) (cyclophosphamide 150 mg/m2 every 12 h on days 1–3, dexamethasone 20 mg/day on days 1–4 and 11–14, and vincristine 2 mg flat dose on days 1 and 8, alternating with methotrexate 250 mg/m2 on day 1 and cytarabine 0.5 g/m2 every 12 h on days 2 and 3 [55, 56]. INO was administered on day 3 of cycles 1 through 4. INO was given as 1.3 mg/m2 for cycle 1 followed by 1 mg/m2 for cycles 2 to 4 (the details of the schedules and doses were summarized in the tables of the reference [81]). Investigators started ursodiol 300 mg three times daily as VOD prophylaxis later as protocol amendment. Maintenance therapy was given as per POMP regimen (for details of the regimen, see references [55, 59, 81]). ORR was 78% (59% CR) with MRD negative rates of 52% (at time of morphological response) and 82% (at 3 months). OS rate at 1 year was 46% (mOS of 11 months). mOS was noted to be higher in patients treated as first salvage regimen (mOS approaching 17 months) compared to those receiving as second salvage regimen. VOD was observed in 6/26 (23%) patients who underwent subsequent AlloSCT and 3/33 (9%) in those who did not receive AlloSCT. All VOD cases had received clofarabine based conditioning regimens with or without busulfan. For patients who are candidates for AlloSCT, treatment with INO should be limited to 2 cycles of induction or the fewest number of cycles required to achieve a CR/CRi (if CR/CRi not achieved after 2 cycles) [55, 79, 80].

### Clinical trials of inotuzumab ozogamicin in NHL

Preclinical Studies confirmed the potency and dose-dependent cytotoxicity of INO on CD22 positive B-lymphoma cell lines and anti-tumor efficacy in mouse models with B-cell lymphomas [72, 73]. When combined with rituximab, additive anti-tumor activity with superior efficacy was achieved in vitro on human B-lymphoma cell lines [82].

Phase 1 studies of INO monotherapy determined maximum tolerated dose (MTD) of 1.8 mg/m2 every 4 weeks in humans with grade 3 or higher thrombocytopenia and neutropenia as the dose-limiting toxicities (DLT). VOD was reported in patients post autologous stem-cell transplant setting and those with prior history of VOD like syndrome [83, 84]. Phase 1/2 study of INO in combination with rituximab (375 mg/m2) every 4 weeks determined MTD of 1.8 mg/m2 every 4 weeks and showed ORR of 87, 74 and 20% in relapsed follicular lymphoma (FL), relapsed DLBCL and refractory B-NHL respectively. 68% of relapsed FL remained progression free at 2 years with median PFS of 17.1 months in relapsed DLBCL and 1.9 months in refractory disease [85]. Thrombocytopenia (56%; 31% grade 3 or higher) and neutropenia (34%; 22% grade 3 or higher) were the most common adverse events requiring dose modification. Serious adverse events included pneumonia (4%), sepsis (3%) and liver dysfunction (4%).

Similar phase 1 study with the combination of rituximab (375 mg/m2) and standard dose INO (1.8 mg/m2) every 4 weeks was studied in the Japanese population [86]. Nine out of 10 patients experienced grade 3 or higher adverse events including thrombocytopenia, neutropenia, elevated liver enzymes and hypophosphatemia; 5 out of 10 patients discontinued treatment because of these adverse events. Overall respone rate (ORR) was reported at 80% (CR 60%).

INO in reduced dose of 0.8 mg/m2 once every 3 weeks has also been studied in combination with rituximab- based chemo-immunotherapy regimens. Phase 1 study of INO in combination with R-CVP (Rituximab, Cyclophosphamide, Vincristine and Prednisone) determined 0.8 mg/m2 as MTD with DLT of reversible grade 4 neutropenia [87]. ORR of 84% (CR 24%) was reported in MTD cohort along with median PFS of 14.4 months and median OS of 24.5 months (aggressive NHL; NR in indolent NHL).

Another phase 1 study of INO (0.8 mg/m2 every 3 weeks) in combination with R-GDP (Rituximab, Gemcitabine, Dexamethasone, Cisplatin) reported ORR of 53% (CR 20%) in refractory/relapsed B-cell NHL with major toxicities of grade 3 or higher thrombocytopenia (75%), neutropenia (62%) and one case of VOD [88] (Table 1).

### Veno-occlusive disease associated with inotuzumab ozogamicin

VOD as seen with gemtuzumab ozogamicin has been reported with the use of INO in the setting of autologous or allogeneic transplant [55–57, 59, 63, 73, 85]. A retrospective study of 26 patients with refractory ALL received INO followed by AlloSCT. Conditioning regimens consisted of cyclophosphamide, clofarabine, fludarabine, melphalan, thiotepa and total body irradiation [89]. Five patients suffered fatal hepatic VOD at a median of 23 days after SCT. In particular, patients who received conditioning with double-alkylating agents (e.g., high-dose busulfan and cyclophosphamide) may be at especially higher risk of VOD [90]. Splitting INO dosage appears to be useful to minimize VOD [55, 56]. Incorporation of blinatumomab as consolidation in the miniHCVD -INO-blinatumomab regimen increases the time between INO and AlloSCT [55, 56, 59, 81]. This may further decrease the VOD risk.

## Conclusion

Single agent inotuzumab ozogamicin has shown higher response rates and longer duration of remission in direct comparison against intensive chemotherapies for R/R B cell ALL. Incorporation of INO into miniHCVD regimen appears to be effective with less toxicity. Although results from NHL trials have not been as encouraging, further studies are still ongoing (Table 2).

**Table 2:** Ongoing trials of inotuzumab ozogamicin (INO)

Reference	Phase	Disease	Intervention	Recruitment
NCT03441061	2	B-ALL with positive MRD	INO	Recruiting
NCT03677596	4	R/R B-ALL	Investigating lower dose level (1.2 mg/m2/cycle) for those with higher risk for liver toxicity or VOD.	Not yet recruiting
NCT03460522	2	Precursor B-cell ALL in 56–74 years old	INO induction followed by conventional chemotherapy	Recruiting
NCT02311998	1/ 2	Ph + B-ALL and CML-blast phase	Bosutinib plus INO	Recruiting
NCT01925131	1	Acute leukemia of ambiguous lineage, Recurrent Ph + B-ALL, Recurrent Burkitt Lymphoma	INO plus CVP (cyclophosphamide, Vincristine, Prednisone)	Recruiting
NCT03739814	2	Ph negative B-ALL	INO followed by Blinatumomab	Recruiting
NCT03851081	1/ 2	r/r B-ALL	INO plus Vincristine (liposomal)	Not yet Recruiting
NCT01664910	1/ 2	Conditioning regimen for HSCT	INO + plus Rituximab, Bendamustine and Fludarabine	Recruiting
NCT03249870	2	Ph negative B-ALL in 55 years or older	INO plus CVP induction	Recruiting
NCT03610438	2	ALL with positive MRD prior to HSCT	INO	Not yet recruiting
NCT03856216	2	Allogeneic SCT	Pre and Post HSCT INO	Not yet recruiting
NCT01371630	1/ 2	Untreated ALL in 60 years and older	INO plus combination chemotherapy	Not yet recruiting
NCT03150693	3	Newly diagnosed B-ALL in 18–39 years old	INO plus chemotherapy	Recruiting
NCT03094611	2	R/R ALL	Lower dose INO	Recruiting
NCT03488225	2	ALL	INO plus HyperCVAD	Recruiting
NCT01679119	2	DLBCL	INO plus R-CVP versus Gem-R-CVP	Recruiting
NCT02981628	2	B-ALL in 1–21 years old	INO	Recruiting
NCT03628053	3	ALL	Tisagenlecleucel versus Blinatumomab or Inotuzumab	Not yet recruiting

*Abbreviations*: *R/R* refractory /relapsed, *CVAD* cyclophosphamide vincristine Adriamycin dexamethasone, *NHL* non-Hodgkin lymphoma, *DLBCL* diffuse large B cell lymphoma, *ALL* acute lymphoblastic leukemia, *Gem* gemcitabine, *R* rituximab, *CVP* cyclophosphamide vincristine prednisone, *VOD* veno-occlusive disease

## Abbreviations

CAR: Chimeric antigen receptor
DLBCL: Diffuse large B cell lymphoma
INO: Inotuzumab ozogamicin
